# Optimal Anesthetic Regime for Motionless Three-Dimensional Image Acquisition During Longitudinal Studies of Adult Nonpigmented Zebrafish

**DOI:** 10.1089/zeb.2016.1343

**Published:** 2017-04-01

**Authors:** Nicola Lockwood, Jennifer Parker, Carole Wilson, Paul Frankel

**Affiliations:** ^1^ Division of Medicine, University College London, London, United Kingdom.; ^2^ CoMPLEX, University College London, London, United Kingdom.; ^3^ Magnus Life Sciences, London, United Kingdom.; ^4^ UCL Fish Facility, Division of Biosciences, University College London, London, United Kingdom.

**Keywords:** zebrafish, husbandry, pigment biology, anesthetic regime

## Abstract

With many live imaging techniques, it is crucial that a deep level of anesthesia is reached and maintained throughout image acquisition without reducing zebrafish viability. This is particularly true for three-dimensional tomographic imaging modalities. Currently, the most commonly used anesthetic in the zebrafish community, MS-222 (tricaine methanesulfonate), does not allow this. We show, using a combination of both MS-222 and isoflurane, that we can significantly improve the anesthetic regime required for motionless image acquisition of live adult zebrafish. We have benchmarked this against the requirements of our novel quantitative imaging platform, compressive sensing optical projection tomography. Using nonpigmented transgenic zebrafish, we show that a combination of 175 ppm of both anesthetics improves the maintenance of deep anesthesia for prolonged periods of time and it can be used repeatedly to enable longitudinal imaging. Importantly, it does not affect the health or viability of the adult zebrafish. We also show that nonpigmented fish, with a mutated form of the gene *transparent*, took significantly longer to reach deep anesthesia. The anesthetic regime presented in this study should lead to significant improvements in accuracy and information achievable from imaging live adult zebrafish and in its application to longitudinal studies.

## Introduction

Although much has been learned from *in vitro* studies, the absence of integrative organ systems and true cellular environments limits their relevance and utility when studying disease and aiding drug development. *In vivo* studies can overcome this. Performing live global imaging of model organisms can provide spatial and temporal information of dynamic interactions at the molecular and signaling level.^1^ Furthermore, it enables longitudinal studies that can provide greater accuracy when studying disease and drug efficacy, while reducing the number of animals required for research.

The use of zebrafish (*Danio rerio*) as a model organism is increasing owing to numerous beneficial features, including a rapid generation time, ease of genetic manipulation, high-throughput capacity, and a fully sequenced, conserved genome.^2^ In addition, the embryonic transparency and generation of nonpigmented mutant lines that lack pigmentation, such as *casper* (*mitfa^w2/w2^*; *roy^a9/a9^*) and TraNac (*mitfa^w2/w2^*, *mpv^b18/b18^*), enable fluorescence detection at all life stages when using optical imaging techniques.^3–6^

We have recently developed compressive sensing optical projection tomography (CS-OPT), a novel three-dimensional (3D) imaging modality capable of longitudinal quantitative analysis of tumor progression and vascular development in live adult nonpigmented zebrafish.^7^ CS-OPT facilitates mesoscopic fluorescence imaging of transparent samples with low phototoxicity and quick acquisition times.

For many longitudinal imaging techniques, it is important that zebrafish reach a deep level of anesthesia, where they have no response to external stimuli, for prolonged periods of time without resulting in detrimental or lethal side effects (Table 1).^8,9^ This is particularly true for CS-OPT, whereby the 3D reconstruction algorithms are reliant upon motionless specimens and any slight movement during acquisition introduces errors in the reconstructed images and resulting quantifications.^5,10,11^

**Table 1.** Definitions of the Levels of Anesthesia Experienced, with Corresponding Physical Movement and Reaction to External Stimuli

*Level*	*Movement*	*Opercula*	*Reaction to stimuli*
Light anesthesia	Equilibrium loss	Increase	Only to substantial
	Erratic swimming		
Deep anesthesia	None	Decrease	None
Medullary collapse (death)	None	Ceases	None

Table adapted from McFarland (1959) and Ross and Ross (2008).

The anesthetic regime widely used by the zebrafish community is 4.2% (168 ppm) MS-222 (tricaine methanesulfonate), based upon its reliability and consistency compared to other agents.^12,13^ However, with prolonged exposure, zebrafish experience respiratory and cardiac failure leading to higher mortality rates.^14,15^ It has been reported that a combination of MS-222 with isoflurane reduces cardiac failure and prolongs time under deep anesthesia before reaching respiratory arrest and medullary collapse.^16^ Furthermore, the recovery period when using the combined anesthesia is reduced when compared to MS-222 alone. However, neither the repeated use of the combined regime nor the maintenance of deep anesthesia was analyzed.

In this study, we report the use of a combined MS-222 and isoflurane anesthetic regime for the immobilization and imaging of the transgenic (Tg) and mutant zebrafish line: TraNac *Tg (KDR:mCherry:Fabp10-rtTA:TRE-eGFPKRASV12).*^7^ This zebrafish line, hereafter referred to as “TraNac transgenic,” is nonpigmented and has a red fluorescent vasculature with an inducible, eGFP-tagged oncogenic KRAS transgene. Upon treatment with the inducer, doxycycline, eGFP-KRASV12 is expressed specifically in hepatocytes leading to tumor progression, which is highly similar to hepatocellular carcinoma.^7^ CS-OPT imaging of adult tumor-burdened fish requires ∼3.5 min to acquire signals for both the tumor and vasculature. This translates to an average time of ∼10 min, when including specimen positioning on the CS-OPT system. We have routinely observed that using MS-222 alone “TraNac transgenic” zebrafish take extended periods of time to reach deep anesthesia in comparison to wild-type (AB) zebrafish and, on occasion, are unable to maintain the lack of response to external stimuli, a characteristic of deep anesthesia (Table 1). We report, in this study, an optimized regime combining doses of MS-222 and isoflurane to enable deep anesthesia that can be maintained for prolonged periods of time. We show that this regime can be repeatedly applied to adult zebrafish without having an impact on health or viability, highlighting its use for longitudinal studies. Furthermore, nonpigmented zebrafish are seen to reach deep anesthesia quicker, thereby reducing the amount of stress experienced.

## Materials and Methods

### Zebrafish husbandry

Zebrafish lines were bred and maintained within the UCL fish facility according to UCL protocols where all procedures conformed to ASPA 1986. System water used had the following parameters: 0 mg/L (undetectable trace) ammonia, under 0.3 mg/L nitrite, under 50 mg/L nitrate, pH 7–8, conductivity of ∼420 microSiemens, 27°C–28°C water temperature, 6 mg/L dissolved gases, and a gH of 4°dGH. The water runs through a recirculating system at a tank flow rate of 6 water changes per hour with a 10% total water change per 24 h. Fish were fed twice daily with both decapsulated Instar I *Artemia* and 2 commercially available microencapsulated diets containing a high crude protein of 40%–50%, which comprised fish meal, soybean, cuttlefish, and wheat in varying quantities. Sexually mature fish were kept at a maximum of 5 per liter and all fish used were female and between 6 and 9 months old.

### Zebrafish strains

Wild-type (AB) zebrafish were held in a colony size of ∼550 and generated from >40 individual pairwise crosses to create the next generation. The strain has been kept at UCL in excess of 16 years as a closed colony. Generation time is between 6 and 9 months.

*Casper* (*roy^−^/roy^−^; nacre^−^/nacre^−^* double mutant) zebrafish were gifted by Leonard Zon (Harvard Medical School) and held as a colony of 80, generated from multiple pairwise crosses and occasional outcrossing to AB, followed by backcrossing to maintain line viability. These have been kept at UCL for ∼10 generations. Generation time is between 9 and 12 months.

TraNac (*tra^−^/tra^−^; nacre^−^/nacre^−^* double mutant) gifted by Julian Lewis (Cancer Research Institute) were held at a colony size of ∼100 as closed colony embryos taken from multiple individual pairwise crosses to create the next generation. These fish have been kept at UCL for approximately four generations. Generation time is between 6 and 9 months.

The transgenic (Tg) line *Tg (KDRmCherry:cmlc2GFP)* was gifted by Ian Zachary (UCL) and they have been held as a colony of ∼30 fish for three generations. They were originally created at UCL and maintained as a sibling incross. They have a wild-type background with mCherry and GFP-labeled vasculature and cardiomyocytes, respectively.

TraNac *Tg (KDR:mCherry:Fabp10-rtTA:TRE-eGFPKRASV12)* zebrafish were held as a colony of ∼30 fish for three generations and maintained as a sibling incross. These were created at UCL through crossing TraNac with both *Tg (KDR:mCherry)* gifted by Steve Wilson (UCL) and *Tg (Fabp10-rtTA:TRE-eGFPKRASV12)* gifted by Zhiyuan Gong (National Univeristy of Singapore). These zebrafish are nonpigmented with mCherry-labeled vasculature and have inducible expression of liver-specific eGFP-tagged KRASV12.

### Anesthetic preparations

A stock solution of 4000 ppm MS-222 (Sigma) was created at pH7 and stored at −20°C, as described in *The zebrafish book.*^12^ Isoflurane (Abbott) was dissolved in ethanol to create a 100,000 ppm stock solution and stored at 4°C. The desired amounts of anesthetic were added to fish water immediately before use.

### Testing anesthesia

Zebrafish were placed into 100 mL of system water containing the prescribed anesthesia concentrations. Deep anesthesia was determined by the loss of righting reflex and response to external stimuli, determined through both tapping the bench and pinching the caudal fin, in line with Table 1. Once this was achieved, the fish were placed into a fluorinated ethylene propylene (FEP) tube used for CS-OPT containing the same anesthetic solution. While in the tube, the fish were closely monitored for movement, response to external stimuli through tapping the bench, and opercula movement. This external stimulus was chosen due to deep anesthesia being the only stage that zebrafish lose their response to vibration or strong pressure, where the latter would be inappropriate to test within the FEP tubing. Furthermore, tapping on the bench is a greater stimulus than what would be experienced during CS-OPT acquisition. This is due to image acquisition being performed upon an optical bench, preventing vibration, and the rotational acceleration being minimal so they are not moved during imaging.^7^ When opercula movement ceased (before medullary collapse) or the fish awoke they were removed from the tube and placed in a recovery tank. Their recovery was monitored and a Pasteur pipette was used to gently squirt oxygenated water over the gills. One hundred percent of zebrafish recovered. Recovery was defined as the ability for the fish to swim once the righting reflex had returned.

### Determining the time to reach deep anesthesia using 168 ppm MS-222 within different zebrafish strains

The strains used for testing MS-222 alone were wild type (AB), *Tg (KDRmCherry:cmlc2GFP)*, Casper, TraNac, and TraNac *Tg (KDR:mCherry:Fabp10-rtTA:TRE-eGFPKRASV12)*. For each strain, *n* = 5 and the statistical tests used were as described below.

### Using a combination of MS-222 and isoflurane to achieve and maintain deep anesthesia

Anesthetic doses trialed included 50/50 ppm (MS-222/isoflurane), 60/60, 65/65, 85/85, 100/100, 150/150, 170/170, 175/0, 175/175, 175/200, and 175/250 ppm. TraNac *Tg (KDR:mCherry:Fabp10-rtTA:TRE-eGFPKRASV12)* zebrafish were used where *n* ≥ 4 for each anesthetic dose.

### Repeatedly using a combination of MS-222 and isoflurane to consistently achieve and maintain deep anesthesia

Anesthesia was repeatedly tested once a week for 4 weeks on TraNac *Tg (KDR:mCherry:Fabp10-rtTA:TRE-eGFPKRASV12)* zebrafish, where *n* ≥ 3 for each anesthetic dose. The anesthesia doses used were 175/175 and 175/200 ppm (MS-222/isoflurane).

### Determining the time to reach deep anesthesia using 175 ppm MS-222 in combination with 175 ppm isoflurane within different zebrafish strains

The strains used were wild type (AB), TraNac, and TraNac *Tg (KDR:mCherry:Fabp10-rtTA:TRE-eGFPKRASV12)*. For each strain, *n* = 5 and the statistical tests used were as described below.

### Statistical analysis

Statistical analysis between numerous groups was performed by one-way analysis of variance (ANOVA) followed by the Student-Newman-Keuls test, where *p* < 0.05 was considered significant.

## Results

### Time to reach deep anesthesia is significantly longer in nonpigmented TraNac fish

We have routinely noticed that “TraNac transgenic” fish require longer exposure to MS-222 to reach deep anesthesia than wild-type (AB) zebrafish. The “TraNac transgenic” fish are nonpigmented, due to lacking both functional copies of the genes *transparent* (*tra)* and *nacre*, and contain two transgenes, KDR:mCherry and eGFPKRASV12. We therefore compared the time to reach deep anesthesia using 168 ppm MS-222 (4.2%) in these fish to other zebrafish genotypes to clarify and elucidate the phenomenon. The genotypes used for comparison included wild type (AB), another double transgenic line [*Tg (KDRmCherry:cmlc2GFP)*], and two nonpigmented zebrafish lines, Casper and TraNac. Casper zebrafish are identical in appearance to TraNac and also share the loss of functional *nacre*, however, they lack both functional copies of the gene *roy.*

**FIG. 1.**
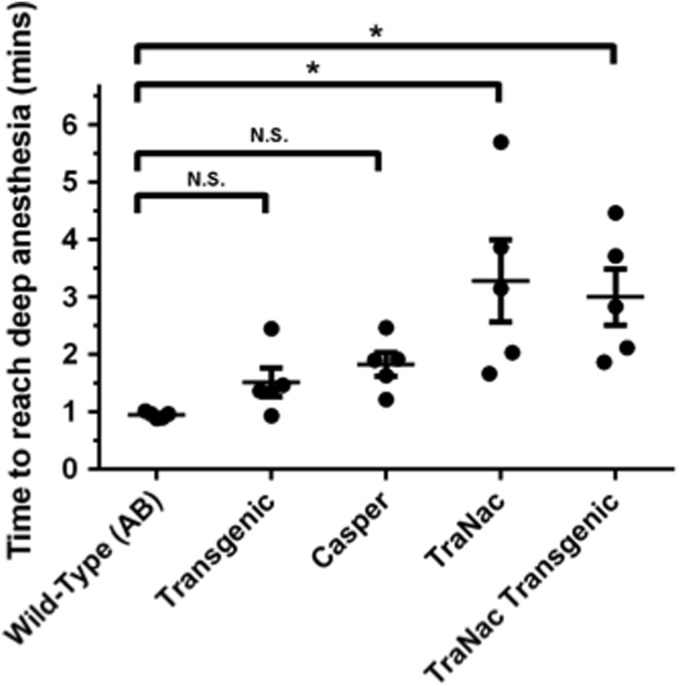
Analysis of the time to reach deep anesthesia within different zebrafish lines using MS-222. The groups of zebrafish tested were wild type **(**AB**)**, Transgenic [*Tg (KDRmCherry:cmlc2GFP)*], Casper (*roy^−^/roy^−^; nacre^−^/nacre^−^*), TraNac (*tra^−^/tra^−^; nacre^−^/nacre^−^*), and “TraNac Transgenic” [TraNac *Tg (KDR:mCherry:Fabp10-rtTA:TRE-eGFPKRASV12)*]. Within each group, *n* = 5 and the error bars represent the SEM. *p < 0.05 and N.S. indicates not significant. SEM, standard error of the mean.

We found that the time taken to reach deep anesthesia in the wild-type (AB) fish was not significantly different to that of the *Tg (KDRmCherry:cmlc2GFP)* or Casper lines, however, a significant increase was seen in the “TraNac transgenic” and the TraNac zebrafish (Fig. 1). Therefore, we can determine that this was not due to the expression of fluorescent transgenes or the nonpigmented phenotype, but was a result of the TraNac genotype, possibly as a result of loss of the *tra* gene.

We also observed fish, of all genotypes, were responsive to every external stimuli test performed during monitoring. Therefore, despite the use of 168 ppm MS-222 allowing deep anesthesia to initially be reached, it was not maintained subsequent to placement within the FEP tube.

### Extended periods of deep anesthesia can be achieved and maintained using combined doses of MS-222 and isoflurane

**FIG. 2.**
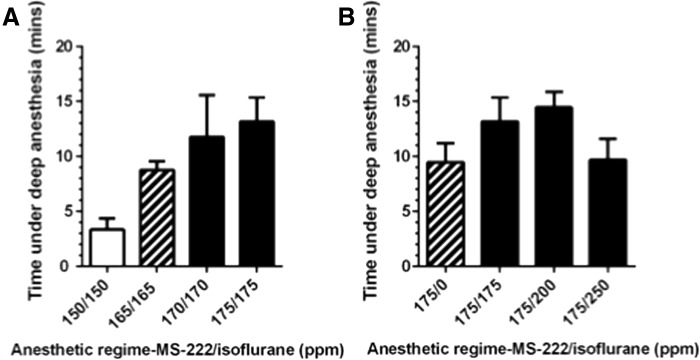
Analyzing time under deep anesthesia using combinations of MS-222 and isoflurane (MS-222 ppm/isoflurane ppm) on “TraNac Transgenic” [TraNac *Tg (KDR:mCherry:Fabp10-rtTA:TRE-eGFPKRASV12)*] zebrafish. Equal doses of the anesthetics **(A)** and variations of isoflurane concentration with 175 ppm of MS-222 **(B)** were analyzed. The data presented for each group are the mean with error bars representing the SEM, where *n* ≥ 4 for each group. *Solid white bars* represent >50% of fish awakening, *hatched bars* represent >50% fish experiencing light anesthesia, and *solid black bars* represent where at least 80% of fish remained under deep anesthesia.

Based upon a previously reported study by Huang *et al.* (2010), “TraNac transgenic” zebrafish were anesthetized with combined doses of MS-222 and isoflurane, in the hope to achieve and maintain longer periods of deep anesthesia (Fig. 2A). In line with Huang *et al.* (2010), we began with equal anesthetic concentrations as low as 50/50 ppm (MS-222/isoflurane); however, all fish exhibited only light anesthesia (Table 1) after 15 min of exposure (data not shown). We next performed a dose escalation with equal amounts of each anesthetic and found that the minimum concentration of MS-222 and isoflurane required for 100% of fish to reach deep anesthesia (Table 1) within 15 min was 150/150 ppm (Table 2). However, despite reaching deep anesthesia, 75% of fish maintained in 150/150 ppm awoke within 5 min of treatment (Fig. 2A). Using a dose of 165/165 ppm resulted in all fish staying under anesthesia, yet 60% were seen to respond to external stimuli suggesting a state of light anesthesia (Table 1). This was significantly improved when using doses of 170/170 or 175/175 ppm, where more than 80% of fish were maintained under deep anesthesia with no response to external stimuli (Fig. 2A and Table 1).

**Table 2.** The Percentage of “TraNac Transgenic” [TraNac *Tg (KDR:mCherry:Fabp10-rtTA:TRE-eGFPKRASV12)*] Fish to Reach Deep Anesthesia Within 15 Min of Exposure to the Combined MS-222 and Isoflurane Anesthetic Regime

*Anesthetic regime—MS-222/isoflurane (ppm)*	*Percentage of fish*
50/50	0
60/60	0
65/65	20
85/85	20
100/100	25
150/150	100

Within each group *n* ≥ 4.

To determine whether longer periods of deep anesthesia could be achieved and maintained, we varied the amount of isoflurane with a constant 175 ppm MS-222, as we reasoned that MS-222 doses higher than 175 ppm would be detrimental to zebrafish due to a concentration of 200 ppm being routinely used as an overdose for zebrafish euthanasia.^14^ Our results show that using 200 ppm of isoflurane (175/200 ppm) had little effect on the time under deep anesthesia compared to 175/175 ppm, while the combination of 175/250 ppm started to trend toward a reduced time (Fig. 2B). Due to this, higher concentrations of isoflurane were not tested and it is possible that the reduced time before opercula cessation was due to toxicity. Using a regime of 175 ppm MS-222 alone resulted in a reduced time under deep anesthesia, with a corresponding increase in response to external stimuli as we have previously observed for 168 ppm MS-222 (Fig. 2B).

Importantly, no significant differences in recovery time were observed using the combined regimes described (Fig. 2B), compared to MS-222 alone (Supplementary Table S1; Supplementary Data are available online at www.liebertpub.com/zeb).

### Repeated use of combined MS-222 and isoflurane consistently achieves and maintains deep anesthesia

**FIG. 3.**
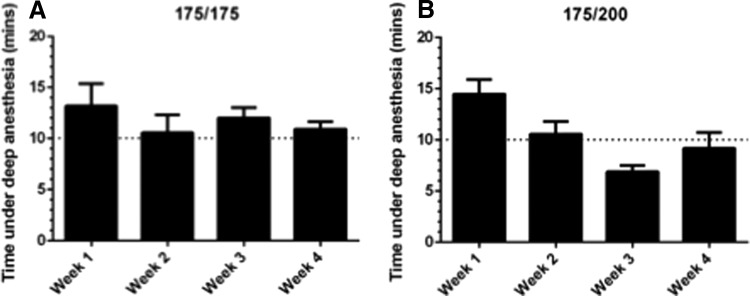
Time under deep anesthesia with repeated use of MS-222 and isoflurane combinations, where **(A)** is 175/175 and **(B)** is 175/200 (MS-222 ppm/isoflurane ppm) on “TraNac Transgenic” [TraNac *Tg (KDR:mCherry:Fabp10-rtTA:TRE-eGFPKRASV12)*] zebrafish. Each anesthetic regime was tested over 4 weeks. The data presented for each group are the mean with error bars representing the SEM, where *n* ≥ 3 for each group. The *horizontal dotted line* at 10 min represents the average time required for CS-OPT image acquisition. CS-OPT, compressive sensing optical projection tomography.

To implement longitudinal studies, repeated anesthesia is necessary. For CS-OPT imaging, one imaging session per week is sufficient to see significant changes in vascular development and remodeling.^7^ Based on results from Figure 2B, we subjected groups of “TraNac transgenic” fish to the combined doses of 175/175 and 175/200 ppm of isoflurane with MS-222, once a week for 4 weeks (Fig. 3). We found that the time under deep anesthesia varied over the 4-week study when using a dose of 175/200 ppm of isoflurane, which was not seen when using 175/175 ppm (Fig. 3). Therefore, we determined that the 175/175 ppm-combined anesthetic regime would be optimal for motionless 3D image acquisition during longitudinal studies. Importantly, repeated exposure to these combined doses of MS-222 and isoflurane did not impact the health, fitness, or viability of the zebrafish. All fish experienced rapid recoveries, did not have reduced viability, or appear under any stress (Supplementary Table S2).

Of note, we found that individual fish that responded slower to anesthesia or maintained opercula movement for longer periods of time tended to exhibit this response over the course of the study (data not shown).

### Applying 175 ppm of both MS-222 and isoflurane reduces the time to reach deep anesthesia

**FIG. 4.**
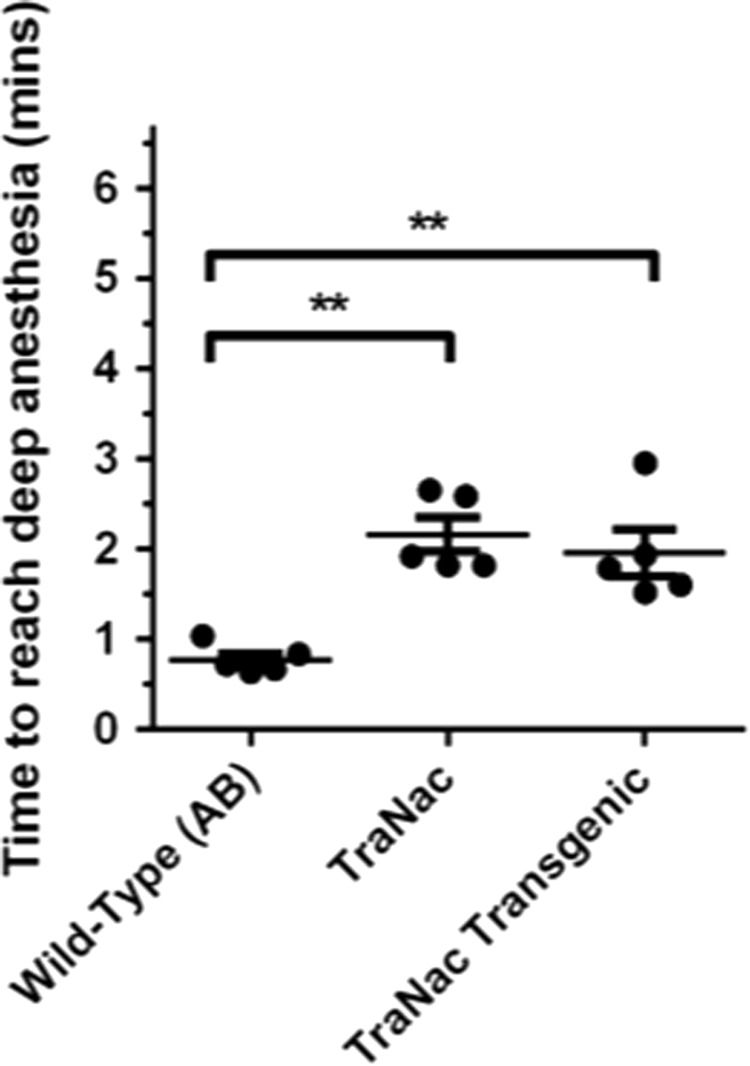
Analysis of the time to reach deep anesthesia within different zebrafish lines using 175 ppm of both MS-222 and isoflurane. The groups of zebrafish tested were wild type **(**AB**)**, TraNac (tra-/tra-; nacre-/nacre-), and “TraNac Transgenic” [TraNac *Tg (KDR:mCherry:Fabp10-rtTA:TRE-eGFPKRASV12)*]. Within each group, *n* = 5 and the error bars represent the SEM. **p < 0.01.

We tested the combined 175/175 ppm of MS-222 and isoflurane on wild-type (AB), TraNac, and “TraNac transgenic” fish to determine whether the time to reach deep anesthesia compared to standard MS-222 alone in different genetic backgrounds was affected (Fig. 4).

All fish were found to reach deep anesthesia in less than 3 min. TraNac and “TraNac transgenic” fish have a reduction in both the time taken to reach deep anesthesia and the variation between individual fish when using the 175/175 ppm-combined regime compared to standard MS-222 used in Figure 1. A significant difference is still observed between the wild-type (AB) group and the TraNac fish, indicating a similar role effect as observed in Figure 1.

## Discussion

In this study, we have repeatedly anesthetized our “TraNac transgenic” zebrafish using a combined dose of MS-222 and isoflurane to quickly reach and maintain a deep level of anesthesia for extended periods of time. This overcomes the shortfalls encountered when using MS-222 alone for 3D imaging of live, nonpigmented adult zebrafish, using modalities such as CS-OPT.

It is known that with prolonged anesthesia with MS-222 alone, zebrafish are susceptible to both respiratory and cardiac failure.^14,15^ It has previously been reported^16^ that a combined dose of 65 ppm of both MS-222 and isoflurane in adult wild-type zebrafish is optimal to improve the time under deep anesthesia without impacting upon survival. Our results show that this dose was not sufficient to induce deep anesthesia in either wild-type zebrafish or in our “TraNac transgenic” fish within 15 min. We did find an improvement using a combination of both MS-222 and isoflurane, however, the minimal dose required for deep anesthesia to be reached and maintained was 150 ppm of both MS-222 and isoflurane.

The combination of isoflurane with MS-222 improves the maintenance of deep anesthesia within our “TraNac transgenic” zebrafish at doses higher than 170 ppm of each anesthetic, which was determined through the response to an external stimulus greater than that experienced during 3D image acquisition. This is an important advancement for imaging live zebrafish, whereby accurate images and quantifications are dependent upon completely motionless animals during acquisition. Furthermore, there was an increase in the time under deep anesthesia before loss of opercula movement was observed, where the 175/175 ppm dose was optimal. The extended time under deep anesthesia could open up additional avenues for imaging. We have previously achieved spatial and temporal readouts of protein activity using Förster resonant energy transfer biosensors through Florescence Lifetime Imaging (FLIM) OPT in zebrafish embryos.^6,17^ Providing a greater period of deep anesthesia could enable FLIM OPT in live adult zebrafish, providing additional information in physiological and pathophysiological contexts. Importantly, we have also shown that this anesthetic regime can be repeated, and does not affect the health or viability of the adult zebrafish. Again, a combined dose of 175/175 ppm of each anesthetic was shown to be optimal, where it consistently achieved an average time under deep anesthesia of greater than 10 min with little variation throughout. This highlights its potential use for longitudinal imaging studies.

We have also shown that both TraNac and “TraNac transgenic” fish require prolonged exposure to MS-222 to reach deep anesthesia compared to wild-type (AB) zebrafish. This difference is not seen in Casper or other double transgenic fish, suggesting a role for the *tra* gene in regulating the MS-222 response. The mutation present in the *tra* gene results in a truncated form of the mitochondrial protein mpv17, which leads to a reduction in iridophores. It has previously been proposed that the loss of mpv17 function could result in alterations in metabolism,^18^ so it is possible that this could account for the differences in response to MS-222. The combined use of 175 ppm of both MS-222 and isoflurane reduces the time to reach deep anesthesia and does not increase the recovery period when anesthetizing the TraNac and “TraNac transgenic” fish. Therefore, the zebrafish will experience less stress as a result. However, it is important to note a difference in time to reach deep anesthesia is still observed when compared to the wild-type (AB) fish. As a community, it is known that zebrafish strains differ between research institutes, as do environmental conditions, and we have shown that response to anesthesia also differs dependent upon genetic backgrounds. Therefore, we note that there may be a need to perform additional adjustments to the 175/175 ppm anesthetic regime for optimization. However, based on our work in this study, it is clear that using a combination of MS-222 and isoflurane is superior to the widely used 4.2% (168 ppm) MS-222 regime.

Thus, incorporating the combined anesthetic dose of 175 ppm of both MS-222 and isoflurane can provide prolonged periods of deep level of anesthesia required for 3D imaging of adult nonpigmented zebrafish, without impacting upon recovery. Moreover, the ability to perform repeated treatments will significantly improve the accuracy and information achievable from imaging live adult zebrafish in longitudinal studies.

## Supplementary Material

Supplemental data

Supplemental data
