# Supplementary figures and images for: Optimal Anesthetic Regime for Motionless Three-Dimensional Image Acquisition During Longitudinal Studies of Adult Nonpigmented Zebrafish
Source: Zebrafish. 2017 Apr 1;14(2):133–9. doi: 10.1089/zeb.2016.1343 (PMC5385422; doi:10.1089/zeb.2016.1343)

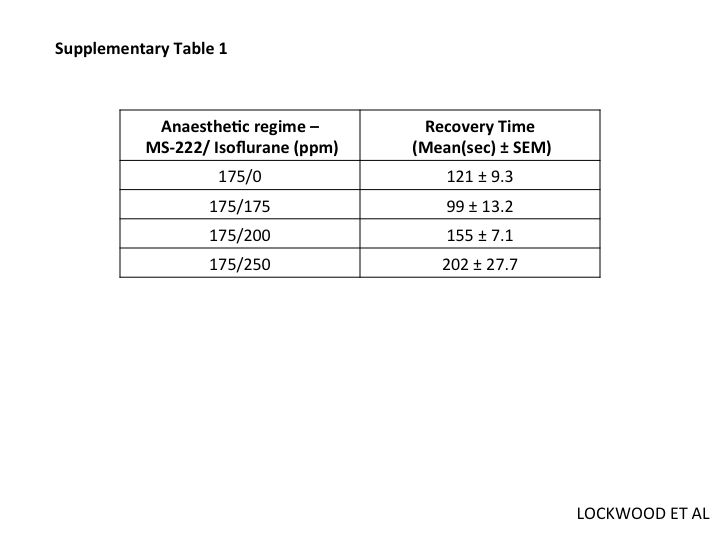

Supplement: Supplemental data [file Supp_Table1.tif]

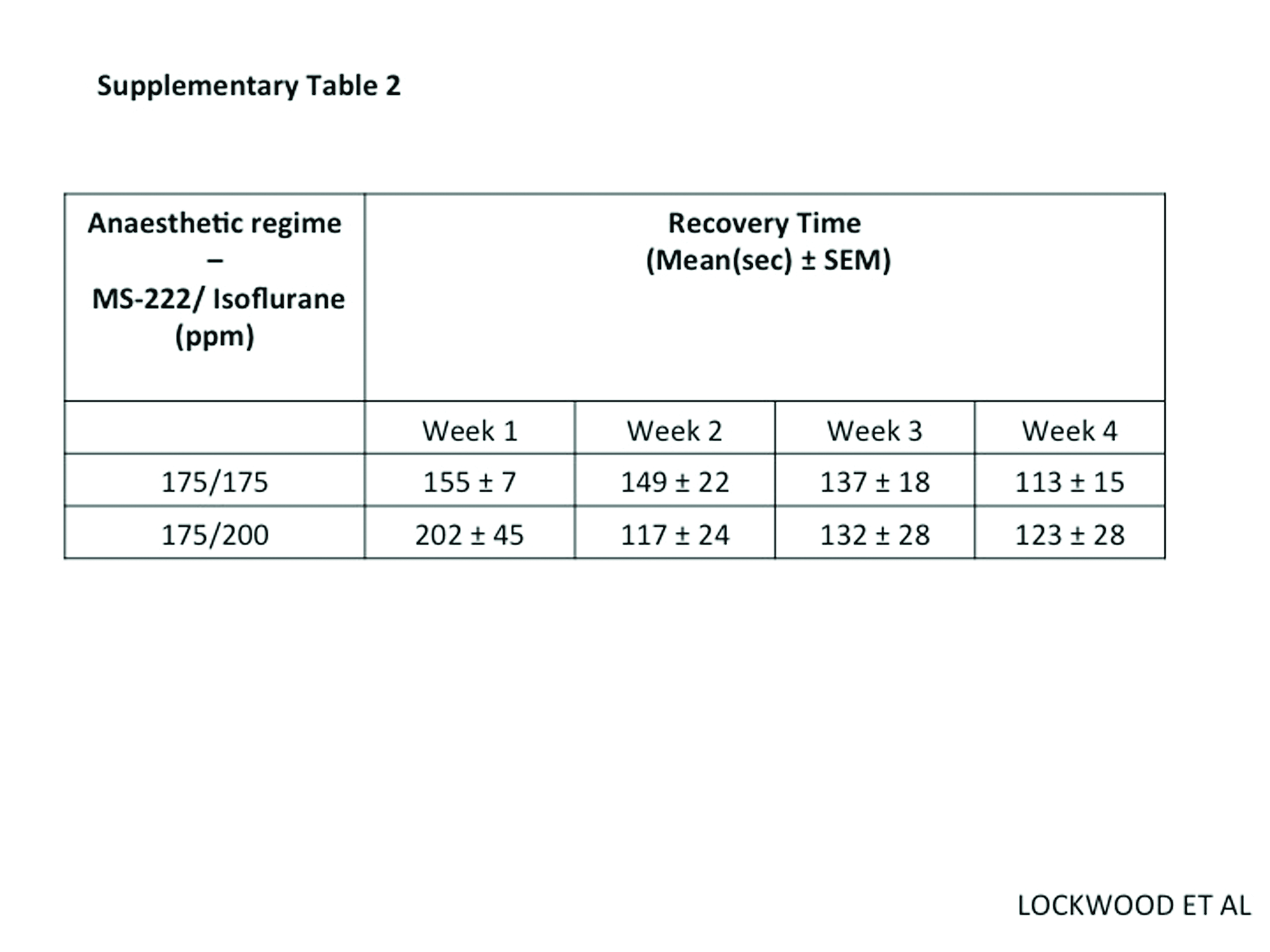

Supplement: Supplemental data [file Supp_Table2.tif]
